# Transactions of the Medical Society of the State of New York

**Published:** 1878-06

**Authors:** 


*Transactions of the Medical Society of the State of New York for the year* 1877. Albany: Van Benthuysen. 1877.

This volume contains many valuable papers which will repay close study. Dr. Parker. in an article upon "Heredity as a Factor in Pauperism and Crime," disagrees with Mr. Dugdale's studies of the "Jukes." He gives as his conclusions: "That as a physiologist I find myself unable to see any heredity as a factor in pauperism, with the exception before stated of the feeble mind and feeble body, and these are rather indirect than direct factors. The environment is, I believe, in this, as in crime, the great factor, and the effort of the State must be to change this radically." Dr. Gouley has formulated his ideas upon the 'Treatment of Stone in the Bladder,' thus: "1st. There is no *best* method of operation or of treatment. 2d. All the operations are good where they are indicated, and bad where they are not. 3d. The operation should be selected for the case, and not the case for the operation." Dr. Burr reports two cases of wounds of the knee joint, without antiseptic treatment, with good results. A similar case was reported in this *Journal* (Vol. XVII. No. 4), in which the result was perfect. The two papers upon closure of cleft of the hard palate, one by Dr. Van Deventer, and the other by Dr. Goodwillie, are interesting and instructive. We have no space to notice the other articles which attracted our attention.

